# Thalamocortical control of propofol phase-amplitude coupling

**DOI:** 10.1371/journal.pcbi.1005879

**Published:** 2017-12-11

**Authors:** Austin E. Soplata, Michelle M. McCarthy, Jason Sherfey, Shane Lee, Patrick L. Purdon, Emery N. Brown, Nancy Kopell

**Affiliations:** 1 Graduate Program for Neuroscience, Boston University, Boston, Massachusetts, United States of America; 2 Department of Mathematics & Statistics, Boston University, Boston, Massachusetts, United States of America; 3 Department of Psychological and Brain Sciences, Boston University, Boston, Massachusetts, United States of America; 4 Department of Neuroscience, Brown University, Providence, Rhode Island, United States of America; 5 Department of Anesthesia, Critical Care and Pain Medicine, Massachusetts General Hospital, Harvard Medical School, Boston, Massachusetts, United States of America; 6 Department of Brain and Cognitive Sciences, Massachusetts Institute of Technology, Cambridge, Massachusetts, United States of America; Université Paris Descartes, Centre National de la Recherche Scientifique, FRANCE

## Abstract

The anesthetic propofol elicits many different spectral properties on the EEG, including alpha oscillations (8–12 Hz), Slow Wave Oscillations (SWO, 0.1–1.5 Hz), and dose-dependent phase-amplitude coupling (PAC) between alpha and SWO. Propofol is known to increase GABA_A_ inhibition and decrease H-current strength, but how it generates these rhythms and their interactions is still unknown. To investigate both generation of the alpha rhythm and its PAC to SWO, we simulate a Hodgkin-Huxley network model of a hyperpolarized thalamus and corticothalamic inputs. We find, for the first time, that the model thalamic network is capable of independently generating the sustained alpha seen in propofol, which may then be relayed to cortex and expressed on the EEG. This dose-dependent sustained alpha critically relies on propofol GABA_A_ potentiation to alter the intrinsic spindling mechanisms of the thalamus. Furthermore, the H-current conductance and background excitation of these thalamic cells must be within specific ranges to exhibit any intrinsic oscillations, including sustained alpha. We also find that, under corticothalamic SWO UP and DOWN states, thalamocortical output can exhibit maximum alpha power at either the peak or trough of this SWO; this implies the thalamus may be the source of propofol-induced PAC. Hyperpolarization level is the main determinant of whether the thalamus exhibits trough-max PAC, which is associated with lower propofol dose, or peak-max PAC, associated with higher dose. These findings suggest: the thalamus generates a novel rhythm under GABA_A_ potentiation such as under propofol, its hyperpolarization may determine whether a patient experiences trough-max or peak-max PAC, and the thalamus is a critical component of propofol-induced cortical spectral phenomena. Changes to the thalamus may be a critical part of how propofol accomplishes its effects, including unconsciousness.

## Author summary

Anesthetics make patients lose consciousness, but how they affect brain dynamics is still unknown. Changes in EEG brainwaves are some of the few noninvasive signals we can use to learn about this. By analyzing such data, we can develop more targeted anesthetics, expand our knowledge of sleep circuits, and better understand how diseases impact these systems. The anesthetic propofol is known, among other effects, to increase synaptic inhibition, but it is unclear how these changes induce EEG alpha (8–12 Hz) oscillations and their interaction with slow wave (0.1–1.5 Hz) oscillations; these signals have been correlated with the state of propofol-infused consciousness. We simulated a network of thalamic cells to understand the mechanisms generating these signals. Propofol-potentiated inhibition produced a novel, sustained alpha rhythm in our network. Changes to the tonic level of depolarization enabled the alpha oscillations to occur at different phases in the slow wave oscillation, as seen clinically with increasing propofol dose. The thalamus may be critical to propofol-induced alpha oscillations and their coupling to slow wave oscillations. By understanding the mechanisms generating alpha, we may be able to design experiments to dissociate alpha from slow waves and determine their independent effects on levels of consciousness.

## Introduction

Propofol is one of the most popular intravenous anesthetics [1]. Despite its ubiquity, the neural mechanisms by which it disables consciousness remain poorly understood [2,3]. Characterization of the critical mechanisms of propofol could enable creation of better targeted anesthetics, identification of consciousness-disabling pathways, and more advanced tools to evaluate depth of anesthesia. This paper presents a novel thalamic sustained alpha rhythm, explores the limits of these thalamic intrinsic oscillations, and describes a thalamocortical origin of propofol phase-amplitude coupling regimes.

**Fig 1:**
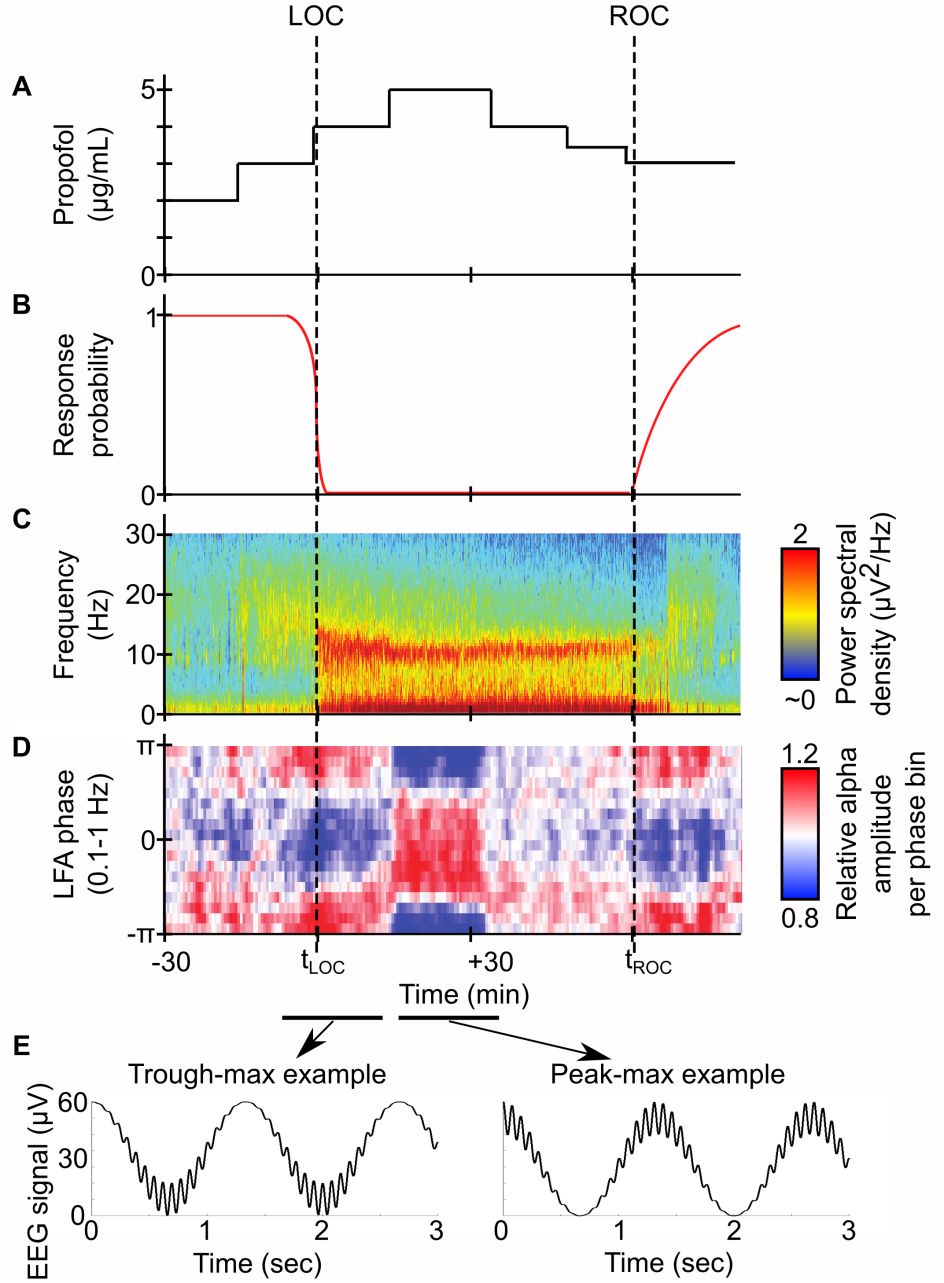
Propofol PAC in EEG profile of stepwise-increasing propofol dose in human volunteers and accompanying illustrations. (A) Illustration of theoretical propofol dose. (B) Illustration of theoretical response profile to auditory cues. (C) Spectrogram of frontal EEG during patient propofol administration. Note the alpha and SWO power between LOC and ROC. (D) PAC modulation of alpha amplitude to SWO phase for same patient. Note the strong, stereotyped trough-max PAC when LOC occurs, and the subsequent 180 degree shift in PAC to peak-max upon deeper propofol administration. (E) Illustration of theoretical EEG traces from representative signals of either PAC regime. Based on Figure 1 of [12].

The many electroencephalogram (EEG) spectral properties of propofol, illustrated in Fig 1, may provide insight into how it accomplishes its effects. Beginning at patient loss of consciousness (LOC), its EEG profile consists of a rise in Slow Wave Oscillation power (SWO, 0.1–1.5 Hz) [2,4–6] and beta power (14–20 Hz) [7,8] that decays to alpha power (8–12 Hz) [3,9,10] for the duration of the anesthesia. Recently, the SWO phase and alpha amplitude were found to exhibit phase-amplitude coupling (PAC) [6,11,12]: around time of LOC, alpha amplitude is maximum at the SWO trough (trough-max), but as the dose increases and the patient experiences deeper anesthesia, the alpha amplitude switches to maximize at the SWO peak (peak-max). How propofol controls alpha-SWO PAC is still a mystery, but investigating the neural mechanisms of propofol-induced spectral properties may allow discovery of the key components needed for LOC provided by propofol.

Propofol rhythms are similar in frequency to sleep rhythms associated with the thalamocortical loop, including SWO [2,13,14] and sleep spindles (10–16 Hz) [9,15]. Mathematical models have played a prominent role in understanding thalamocortical rhythms in both sleep and anesthesia. There is a strong history of modeling SWO [16–21], especially in cortex [22–24], and likewise for thalamic spindle generation [25,26]. More recently, many facets of propofol rhythms have been modeled, including transient propofol beta [8], burst-suppression [27], corticothalamic entrainment of alpha [28], alpha anteriorization [29], and neuromodulatory impacts on propofol SWO [21]. However, the fundamental source of propofol alpha, its relationship to anesthetic hyperpolarization, and its relationship to the GABA_A_ potentiation of propofol are all unknown. Specifically, while we have previously modeled propofol alpha arising from propofol GABA_A_ potentiation and thalamocortical entrainment of this propofol alpha [28], we did not investigate the mechanisms of its creation. Additionally, there have been no modeling studies examining either the mechanistic relationship between SWO and alpha under propofol (including their PAC), why this PAC is affected by dose, or the effects of direct hyperpolarization on these dynamics in this system. Since propofol alpha appears to be sustained over the entire course of anesthesia [11,12,30] and propofol alpha has been detected in thalamic recordings [31–35], the relationship of that rhythm to waxing-and-waning spindle oscillations is also unclear.

To better understand how propofol sustained alpha and alpha-SWO PAC are generated, we simulated a thalamic, Hodgkin-Huxley network model under corticothalamic SWO input. We found that the thalamus is able to generate a sustained alpha rhythm independently of cortical input. While thalamic intrinsic oscillations are sensitive to both thalamocortical (TC) cell H-current maximal conductance (g_H_) and background excitation (excitation from brainstem and cortex) [36,37], only under strong propofol GABA_A_ potentiation did we find a persistent, sustained alpha rhythm. This thalamic sustained alpha may be relayed up to cortex, where it is subsequently seen on the EEG similar to that seen under propofol unconsciousness in the operating room. Our simulations also showed that the thalamus may control the phase of the alpha coupling to SWO. Surprisingly, changes to the background excitation alone could control the PAC regime of the system between trough-max and peak-max. Our analysis of the mechanisms of propofol PAC could help differentiate the roles of thalamus and cortex in propofol phenomena, leading to better understanding of anesthetic action and the components of consciousness.

## Results

### GABA_A_ potentiation enables propofol-induced sustained alpha

**Fig 2:**
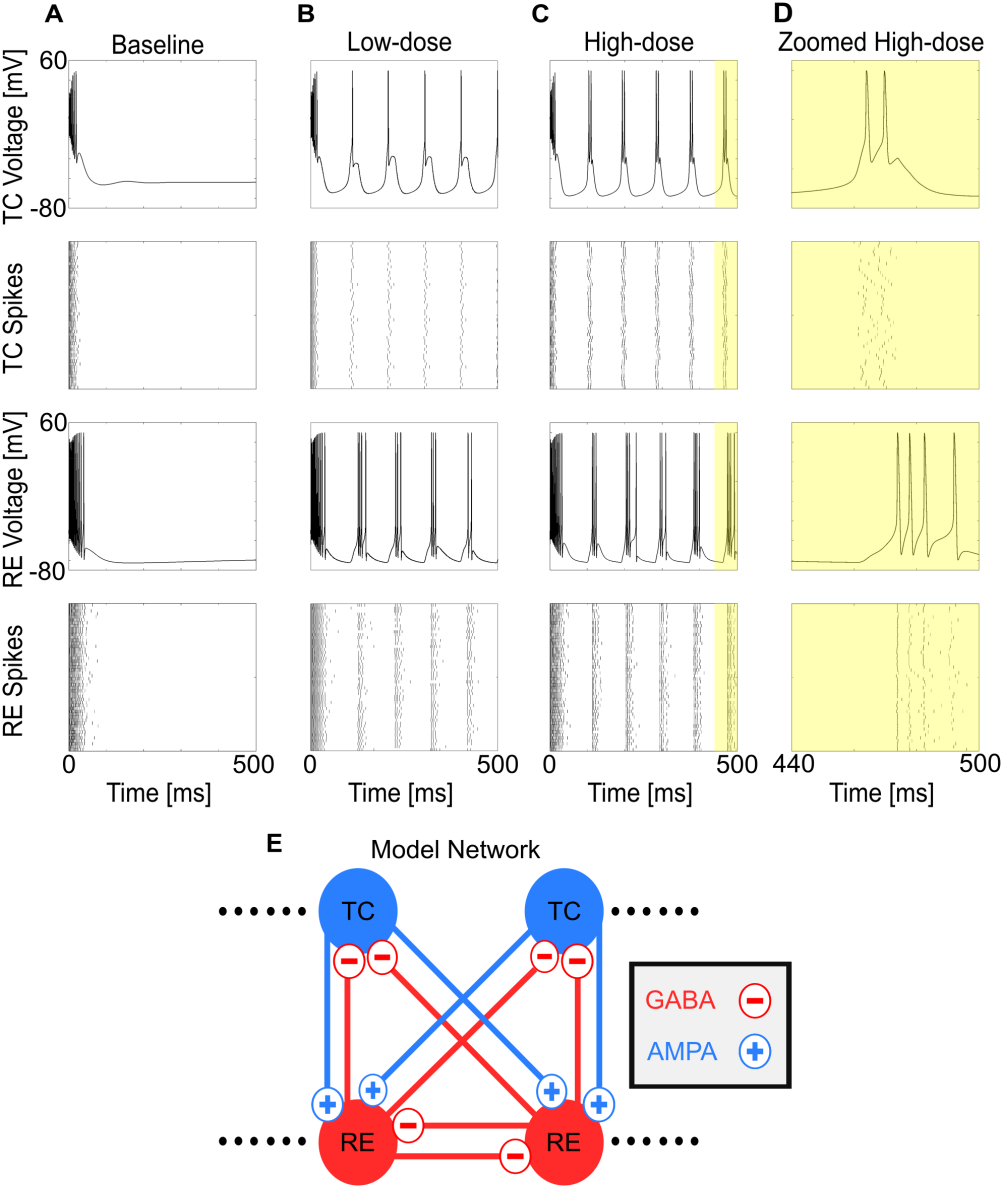
Propofol enables sustained alpha firing in thalamus. (A) Representative voltage traces and spike rastergrams of TC and RE cells under baseline (no propofol) conditions. (B) Representative voltage traces and spike rastergrams of TC and RE cells under the same conditions, except for low-dose propofol potentiation of GABA_A_. (C) Representative voltage traces and spike rastergrams of TC and RE cells under the same conditions, except for high-dose propofol potentiation of GABA_A_. (D) Zoom of C, illustrating time course of a single alpha TC-RE burst. (E) Simplified connection matrix of the 50TC-50RE simulations used.

We began by investigating sustained thalamic alpha; this is the first step in understanding alpha-SWO PAC, in which the alpha rhythm may occur in only parts of the SWO phase. For all simulations, unless otherwise noted, we considered a 50 thalamocortical cell (TC) and 50 reticular cell (RE) computational thalamic model for our thalamus (see Methods), inherited from [28], itself derived from a well-established model [37]. Under "baseline" conditions (no propofol), the model thalamus did not produce any intrinsic oscillatory activity, but instead settled into a stable, silent depolarized state after a transient from initial conditions (Fig 2A). To model low-dose propofol, we doubled both the maximal GABA_A_ conductance, g_**GABAA**_, and the decay time constant of GABA_A_ inhibition, τ_**GABAA**_, throughout the system [8,28]; to model high-dose propofol, we tripled both g_**GABAA**_ and τ_**GABAA**_. Simulating the same baseline system as above, but under either low-dose or high-dose propofol conditions, we found sustained, persistent alpha firing (Fig 2B, 2C and 2D). Synaptic effects from propofol can enable sustained alpha firing in the thalamus, and we explore how this comes from intrinsic properties of the thalamus.

The thalamus is known to display a range of behaviors, largely dependent on the excitation level of the system, TC cell g_H_, and TC cell T-current window interaction: tonic spiking, silent depolarization, hyperpolarized oscillation including spindling and SWO, and silent hyperpolarization [36,37]. This raises the question of whether sustained alpha oscillation can emerge in "normal" thalamus, especially in oscillations near the alpha frequency range, such as spindles. Because both the level of background excitation, also known as applied current, and the level of g_H_ are known to be variable and alter the dynamical state of the thalamus [16,36,37], we simulated across both of these dimensions to search for regions of sustained alpha oscillations. Note that our use of background excitation is not meant to model direct inhibition into the system, but rather the sum of hyperpolarizing effects from changes to neuromodulators, second-order neuromodulatory effects, and loss of brainstem excitation. Since we did not know how weakly or strongly hyperpolarizing the anesthetic was to the system, we modeled the sum of these effects as voltage-invariant tonic charge changes to investigate the entire dimension. Thus, negative background excitation represents tonic hyperpolarization of the system.

**Fig 3:**
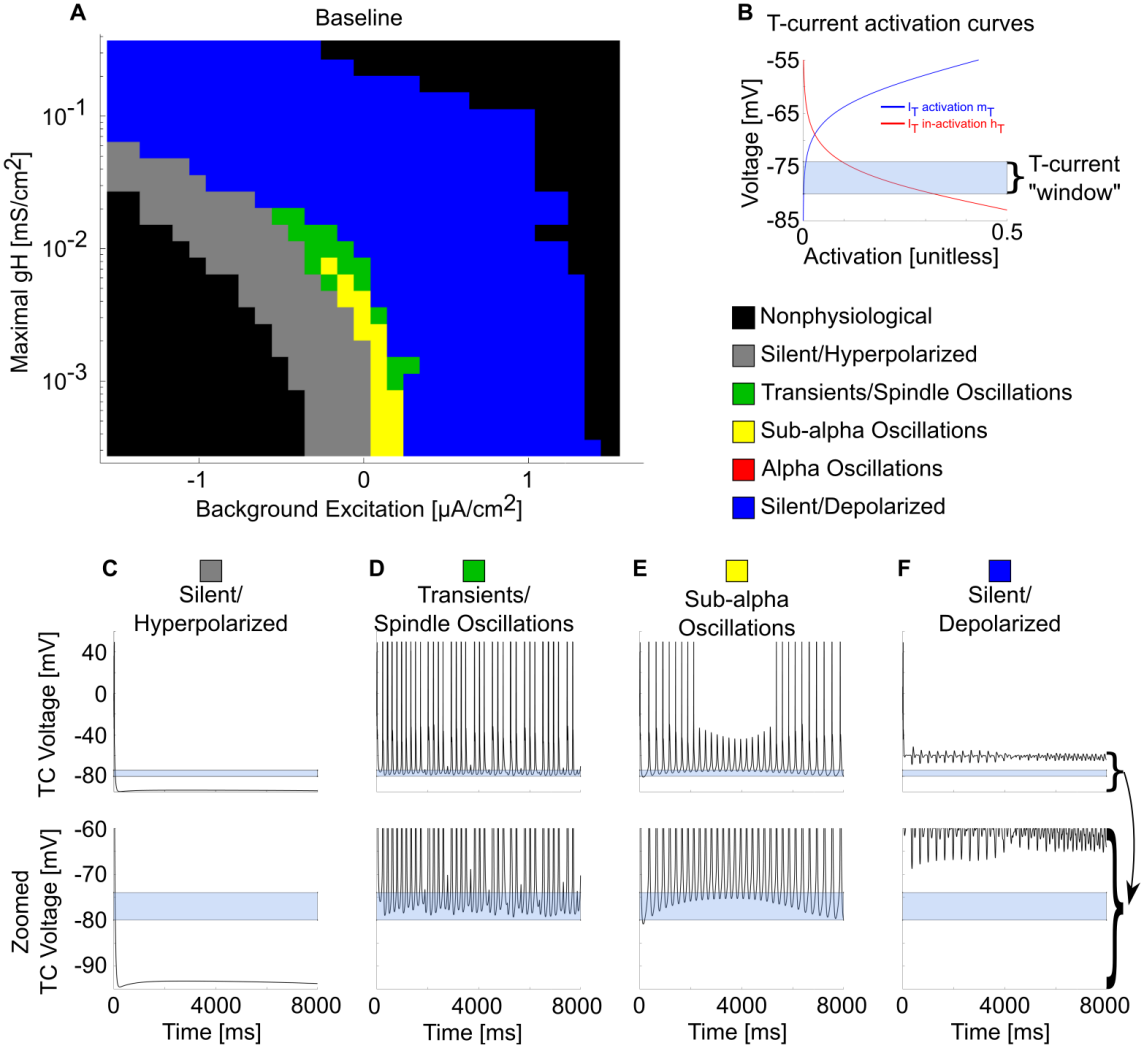
Many thalamic oscillations, but not sustained alpha, occur across the baseline g_H_-background excitation plane. (A) Behavioral regimes of baseline thalamic simulations across all physiological values of the g_H_-background excitation plane. Each simulation is represented by a pixel, colored according to its manually classified behavioral regime. Note the lack of sustained alpha (red) firing. (B) TC cell T-current de-inactivation "window" illustrated by the sum of steady-state activation (mT, blue) and de-inactivation (hT, red) curves. In our model, activation mT is always at steady-state, while the de-inactivation hT state variable must remain high enough for long enough to enable a spiking T-current burst. (C) Representative TC cell voltage trace during a silent, hyperpolarized stimulation and its zoom near the T-current de-inactivation window. (D) Representative TC cell voltage trace during a spindling simulation and its zoom. (E) Representative TC cell voltage trace during a sub-alpha simulation and its zoom. (F) Representative TC cell voltage trace during a silent, depolarized simulation and its zoom.

Over the entire physiological range of the g_H_-background excitation plane, sustained alpha oscillations did not emerge under baseline conditions (Fig 3). However, alpha transients in the form of spindles occurred. These were distinguished from propofol sustained alpha by their waxing and waning nature. Although propofol decreases g_H_ [38], these results indicate that decreasing the H-current alone is not sufficient to enable sustained alpha. Similarly, by varying background excitation to model ascending brainstem neuromodulation, we also found this was not sufficient to enable sustained alpha. Therefore, sustained alpha is likely not a normal thalamic rhythm, and propofol GABA_A_ potentiation is likely to be responsible for generating sustained alpha.

### Propofol dose-dependently increases the dynamic range of sustained alpha oscillations

**Fig 4:**
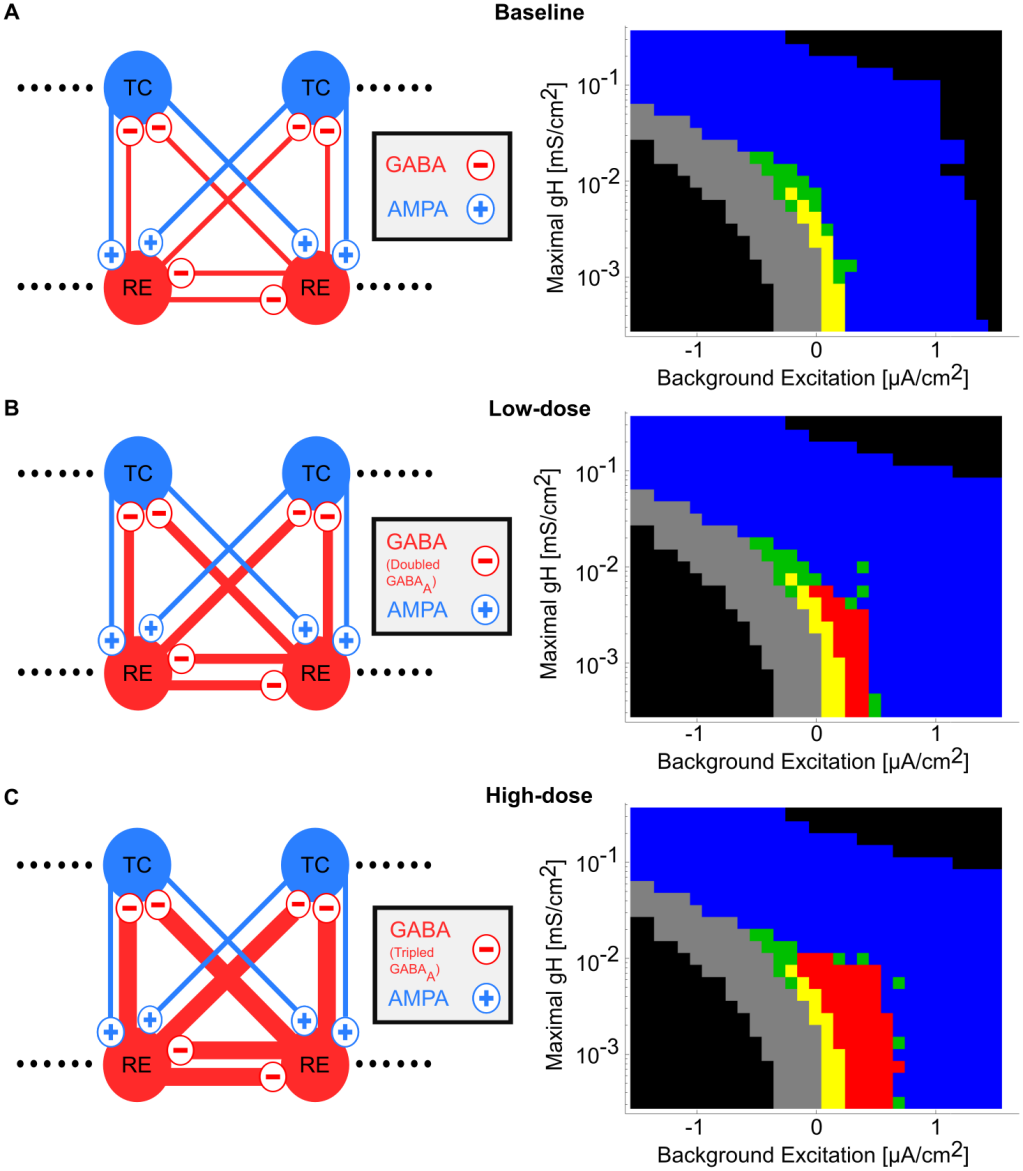
Propofol potentiation of g_GABAA_ and τ_GABAA_ enables sustained alpha oscillations on the g_H_-background excitation plane. (A) Model network and behavioral regimes of simulations across the g_H_-background excitation plane under baseline. See Fig 3 for behavior regime legend. (B) Model network and behavioral regimes of simulations across the g_H_-background excitation plane under low-dose propofol. Note the presence of sustained alpha (red) simulations. (C) Model network and behavioral regimes of simulations across the g_H_-background excitation plane under high-dose propofol.

Our simulations so far suggest that potentiation of GABA_A_ by propofol enables sustained alpha (Fig 2B, 2C and 2D), but we have yet to demonstrate the robustness of these propofol-induced oscillations to changes in g_H_ and background excitation. To do this, we analyzed the behavior regimes of each simulation under low-dose and high-dose propofol across the physiological g_H_-background excitation plane in (Fig 4B and 4C). In baseline simulations in Fig 4A, as background excitation increases, the system shifts from sub-alpha oscillations and transients/spindling into silent depolarization. When propofol is applied in low-dose and high-dose propofol planes (Fig 4B and 4C), increasing the background excitation enables the system to fire in sustained alpha oscillations. The sustained alpha oscillations emerge from the same region of the g_H_-background excitation plane, and even at low-dose propofol it comprised roughly the same area as all other intrinsic oscillations combined.

The size of this sustained alpha "firing area" roughly doubled when the GABA_A_ potentiation of propofol was tripled (high-dose, Fig 4C) compared to doubled (low-dose, Fig 4B). The region of sustained alpha expanded into states where the background excitation was even higher. This effect was due to increasing the dynamic range over which excitation and inhibition were balanced (see next subsection). However, other than this increased propensity for sustained alpha firing, there were no major differences between the sustained alpha oscillations of low-dose versus high-dose propofol. For this reason, when we discuss mechanisms of the sustained alpha, we do not differentiate between sustained alpha coming from low-dose versus high-dose propofol simulations and may only show high-dose simulations in the interest of brevity. Note that, as in the baseline simulations, the g_H_ of the system must be below some threshold, ~0.024 mS/cm^2^, in order for any intrinsic thalamic activity to occur, including sustained alpha. Therefore, it is necessary but not sufficient to decrease g_H_ below this threshold to produce propofol-induced sustained alpha. By applying low-dose or high-dose propofol via, doubling or tripling both g_**GABAA**_ and τ_**GABAA**_, respectively, we find that the synaptic GABA_A_ effects of propofol enable sustained alpha in a dose-dependent manner.

### Propofol induces sustained alpha via changing the balance of excitation/inhibition

**Fig 5:**
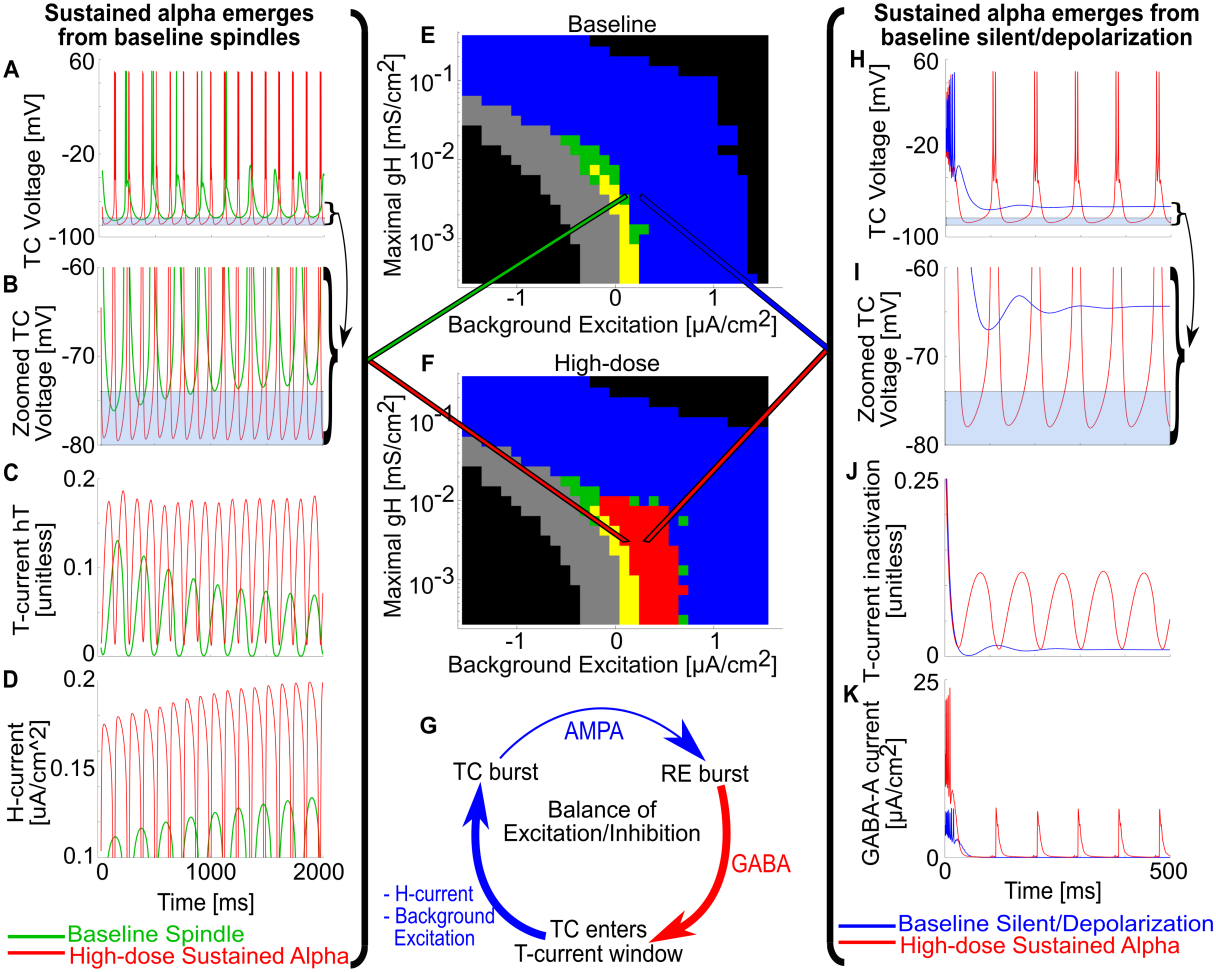
Propofol g_GABAA_ potentiation enables sustained alpha by changing the balance of excitation and inhibition. (A) Representative TC voltage traces for the same g_H_-background excitation state under either baseline spindling (green trace) or high-dose propofol sustained alpha (red trace). (B) Zoom of A around T-current de-inactivation window. Baseline spindles terminate from H-current up-regulation, which raises the minima of TC cell voltage outside the T-current de-inactivation window. Under high-dose propofol g_**GABAA**_ potentiation, the TC cell voltage floor does not rise from the window. (C) Representative TC cell T-current de-inactivation state variables (hT) for baseline and high-dose. Note that baseline spindles stop spiking once the hT maxima are below a threshold. (D) Representative TC cell H-current magnitude of baseline and high-dose. Note that baseline spindles stop spiking once H-current magnitude is high enough, but high-dose sustained alpha continues to spike even with a stronger realized H-current. (E) g_H_-background excitation plane for baseline simulations. (F) g_H_-background excitation plane for high-dose propofol simulations. (G) Illustration of the intrinsic TC-RE oscillation cycle; propofol g_**GABAA**_ potentiation and negative background excitation enhance the RE burst inhibition, while H-current and positive background excitation augment the time from TC voltage minimum to TC bursting. (H) Representative TC voltage traces for the same g_H_-background excitation state under either baseline silent depolarization (blue trace) or high-dose propofol sustained alpha (red trace). (I) Zoom of H around T-current de-inactivation window; baseline never enters the T-current window, and therefore does not oscillate. (J) Representative TC cell T-current de-inactivation state variables for baseline and high-dose. (K) Representative TC cell H-current magnitude of baseline and high-dose.

Having shown that GABA_A_ potentiation by propofol can lead to robust sustained alpha oscillations in the model, we next explored the dynamical mechanisms underlying its production in thalamic networks. As we compare high-dose propofol simulations to their baseline counterparts in Fig 5, there are two main facets to how propofol enables sustained alpha in this thalamic system: engaging thalamic spindling dynamics and balancing enhanced inhibition with excitation.

Propofol takes advantage of intrinsic spindling dynamics in producing sustained alpha. During high-dose propofol, sustained alpha was present where there were either transients/spindles or silent depolarization in the baseline case (Fig 5). In the case of transients/spindling, Fig 5A shows both the voltage traces of a terminating spindle at baseline and sustained alpha at high-dose propofol; these simulations correspond to the same point in the g_H_-background excitation plane and are differentiated only by whether propofol has potentiated GABA_A_ synapses or not. As in prior modeling work [26,37], the spindle at baseline wanes due to Calcium-based up-regulation of the H-current, as can be seen by the increasing magnitude of current in (Fig 5D). At baseline, this slow, depolarizing current raises the baseline TC cell membrane potentials above the T-current activation window such that bursting cannot occur (Fig 5B). In contrast, when GABA_A_ is potentiated by propofol, sustained alpha does not wane, since the RE inhibition consistently hyperpolarizes TC cells into the T-current window, enabling T-current bursts to occur (Fig 5B). Note that the H-current is more active in the high-dose propofol case than at baseline even though the maximal conductance is the same (Fig 5D), yet the H-current is not strong enough to overcome the enhanced RE inhibition of propofol (up to a point of g_H_ > 0.024 mS/cm^2^, see previous subsection). Similarly, in Fig 5H through 5K, propofol enabled sustained alpha oscillations in parameter regimes where, due to the strength of the background excitation under baseline conditions, there would otherwise be silent depolarization. This new sustained alpha occurred for the same reason: the enhanced inhibition forces TC membrane potentials into the T-current window.

The second major facet of propofol-sustained alpha concerns how propofol changes the balance of excitation and inhibition. The fundamental cycle of the excitatory inputs to this system (TC cell H-current and positive background excitation) balanced against the inhibitory inputs (propofol GABA_A_ potentiation and negative background excitation) is illustrated in Fig 5G. TC cells fire and activate RE cells, which burst and have their inhibition onto TC cells magnified by propofol. This greater inhibition paradoxically increases the probability of a TC cell burst by hyperpolarizing the TC cell membrane potentials into the T-current window. Upon entering the T-window for long enough, the T-current de-inactivation state variable (hT) builds up, and time until the next burst is decreased by depolarizing effects such as positive background excitation and TC H-current. The amount of augmenting depolarization affects the frequency of the oscillation, determining if the propofol-infused system oscillates at sub-alpha frequencies like theta or delta, or sustained alpha. The TC bursts again, and the cycle is reset. The enhanced inhibition is also the reason for the dose-dependent increase in sustained alpha area on the g_H_-background excitation plane in Fig 4B and 4C: greater RE inhibition by high-dose vs. low-dose propofol allows high-dose propofol to enable sustained alpha oscillations under a broader range of stronger background excitation.

**Fig 6:**
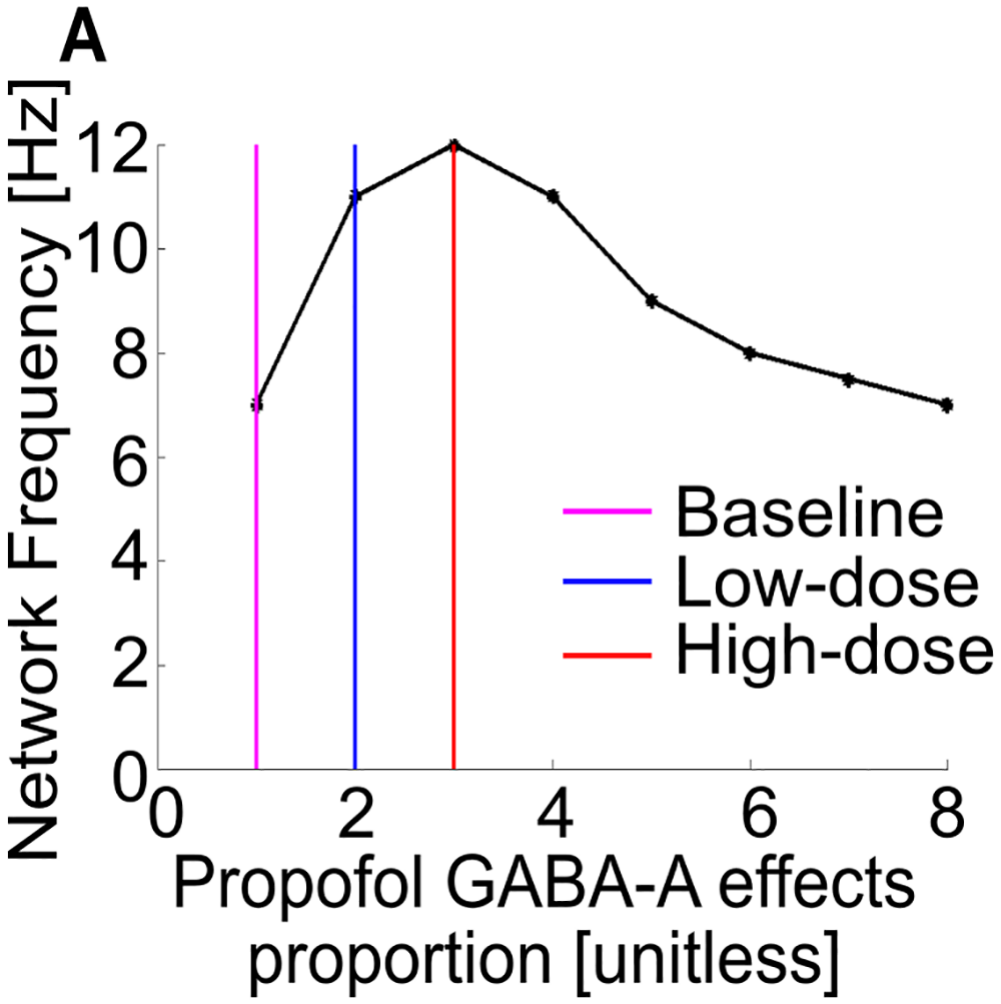
Maximum network frequencies under TC cell propofol multiplier extremes. (A) The maximum network frequency peaks at high-dose levels of g_**GABAA**_ potentiation, where g_**GABAA**_ and τ_**GABAA**_ are tripled. Increasing the g_**GABAA**_ potentiation further to extreme, possibly non-physiological values, only decreases the maximum network frequency.

Increases in propofol from baseline lead to an increase in network frequency up to a maximum (Fig 6); the sustained alpha emerges at the peak, and its frequency decreases with further propofol potentiation. Increases in background excitation increase the thalamic oscillation frequency up to its network frequency maximum, after which the system is too depolarized to oscillate. These results are consistent with alpha being the maximum thalamic frequency possible via hyperpolarized intrinsic oscillations [28].

### The thalamus can produce all PAC regimes under corticothalamic SWO

**Fig 7:**
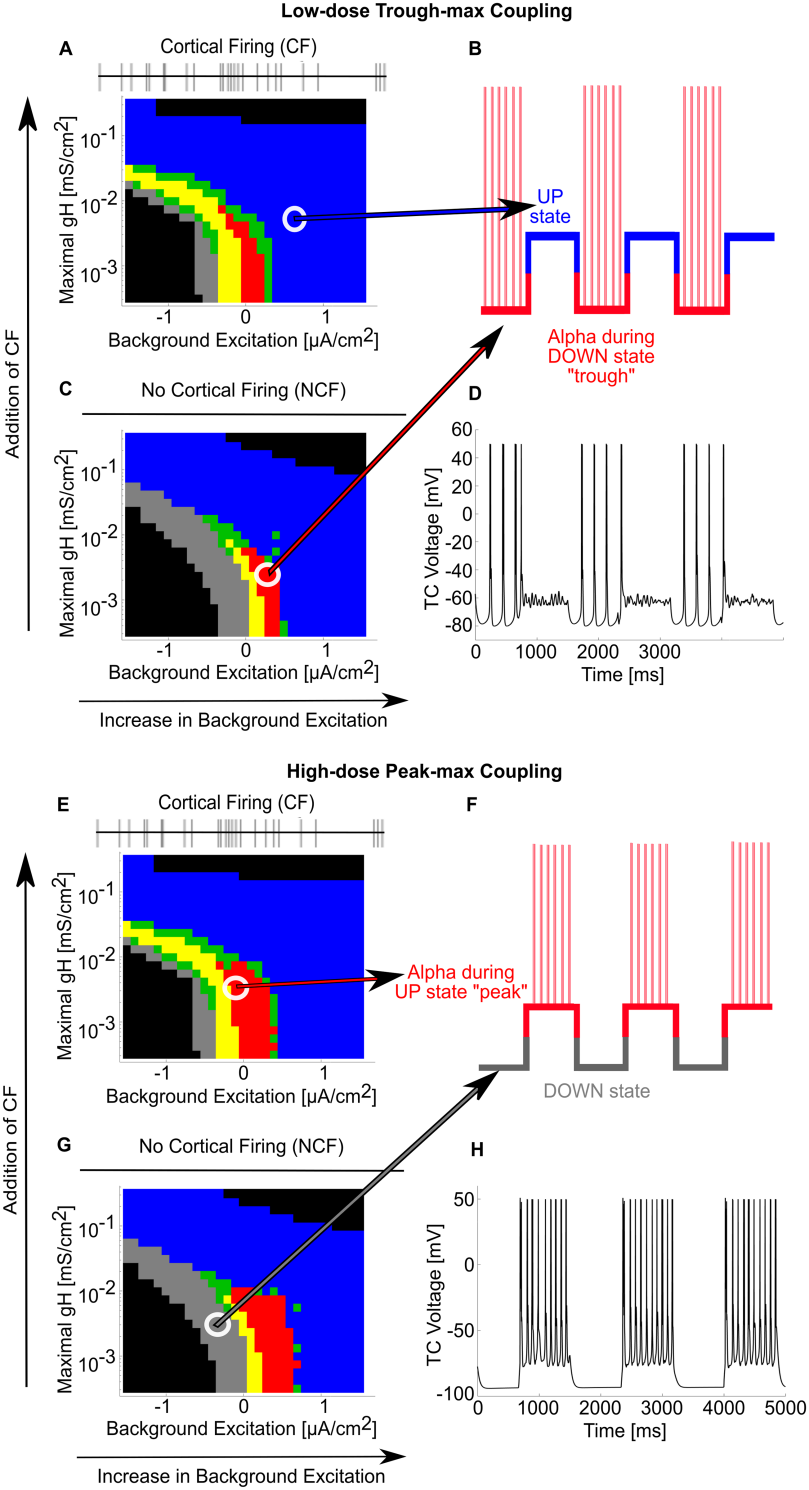
Propofol PAC regimes can be modeled as SWO connections between different g_H_-background excitation planes. (A) Behavioral regimes of simulations across the g_H_-background excitation plane under low-dose propofol with no cortical firing (NCF). See Fig 3 for behavior regime legend. (B) Illustration of where each CF and NCF state lies on the SWO for trough-max: the blue, silent, depolarized CF state occurs during the corticothalamic SWO UP state, while the red, sustained alpha NCF state occurs during the corticothalamic SWO DOWN state. (C) Behavioral regimes simulations across the g_H_-background excitation plane under low-dose propofol with cortical firing (CF). (D) Representative TC cell voltage during SWO oscillation between the two states indicated in (A) and (C), illustrating trough-max. (E) Behavioral regimes of simulations across the g_H_-background excitation plane under high-dose propofol with NCF. (F) Illustration of where each CF and NCF state lies on the SWO for peak-max. (G) Behavioral regimes simulations across the g_H_-background excitation plane under high-dose propofol with CF. (H) Representative TC cell voltage during SWO oscillation between the two states indicated in (E) and (G), illustrating peak-max.

We have shown how propofol enables thalamic sustained alpha, but in order to analyze propofol PAC between alpha and SWO, we constructed a model of corticothalamic SWO UP and DOWN states in the thalamus. UP states have two effects on the thalamus: cortical firing (CF) and a tonic depolarization step, while DOWN states have no cortical firing (NCF) and do not include a tonic depolarization step [14,20]. All of our simulations so far have no spiking inputs and can be used to model NCF states. To model CF, we simulated g_H_-background excitation behavior planes for all three dose levels under the influence of an AMPA-ergic 12 Hz Poisson spiking process [28,39]. The behavior under low-dose and high-dose conditions are respectively shown in Fig 7A and 7E. As a result of introducing CF to the thalamic network, the system oscillates throughout more of the parameter space, and slightly less depolarization is needed to elicit thalamic oscillation, but the CF does not significantly alter the sustained alpha oscillation or overall behavior. Separately, we can model any tonic depolarization steps as increases in background excitation, so the depolarizing step component of the shift from DOWN to UP is accounted for if the system moves to the right on the background excitation axis as in Fig 7A and 7C. Similarly, a hyperpolarizing step from UP to DOWN is identical to moving the state of the system from the right to the left on the background excitation axis as in Fig 7A and 7C. Additionally, as dose increases from trough-max to peak-max PAC, we can model the progressive loss of brainstem excitation simultaneously as an overall decrease in background excitation of both UP and DOWN states. By analyzing the thalamic networks across the g_H_-background excitation plane, propofol dose, and CF/NCF, we can compare UP states to DOWN states, therefore enabling steady-state SWO modeling of the thalamic network.

**Fig 8:**
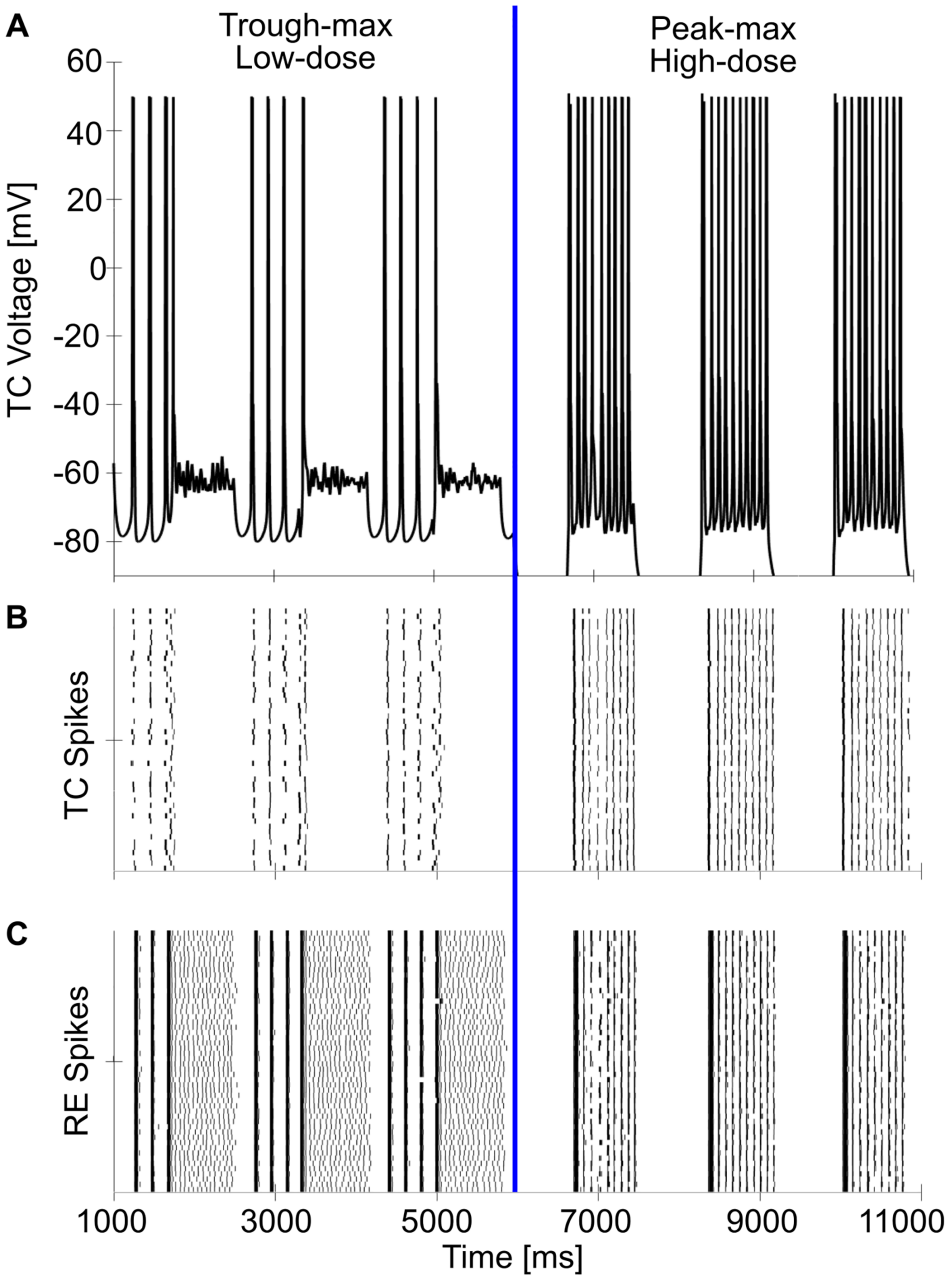
Single simulation illustrating trough-max and the switch to peak-max. (A) Representative TC cell voltage across trough-max and peak-max. At the halfway point (6 seconds), the switch from low-dose to high-dose propofol is simulated by increasing g_**GABAA**_ potentiation from double to triple of baseline and reducing background excitation. (B) Rastergram of the 50 TC cells, illustrating the high synchrony across the network. (C) Rastergram of the 50 RE cells, illustrating their high synchrony.

During trough-max, we found our thalamic model could couple its alpha oscillation to the trough of the cortical SWO input, driving cortical alpha power trough-max PAC (Fig 7A through 7D and illustrated in Fig 8). Under low-dose, trough-max conditions, the thalamic DOWN state is hyperpolarized such that it expresses sustained alpha even though there is no cortical input. Note that this is a change in hyperpolarization through decreasing background excitation, not a change in g_H_. This is illustrated in Fig 7B and 7C, and a simulation is shown in the DOWN states of Fig 7D. Under a thalamic UP state, however, the incoming CF and increase in background excitation depolarize the TC cells out of their sustained alpha regime, forcing them into their silent depolarized state as shown in Fig 7A. Thus, the cortex will receive strong alpha input from the thalamus only during its DOWN states, when the thalamus is independent, and this thalamic alpha input will cease during the UP states, allowing the cortex to experience trough-max PAC.

Similarly, during peak-max PAC, we found the thalamic network could be the alpha driver on the cortical SWO peak (Fig 7E through 7H). As the high-dose thalamus can be more hyperpolarized than the low-dose state, the DOWN state of the thalamus experiences silent hyperpolarization as in Fig 7G. However, upon receiving CF and depolarization from the cortical UP state, the thalamic UP state exhibits sustained alpha output (Fig 7E). The cortex receives no thalamic input during DOWN states but receives sustained alpha thalamic input during UP states, producing peak-max PAC observable in the cortex.

Surprisingly, we also found that a change in only thalamic background excitation could enable switching between trough-max and peak-max PAC. Keeping the state of the system steady on the g_H_-background excitation plane, increasing GABA_A_ potentiation was insufficient to switch between trough-max and peak-max. While UP/DOWN alternation in the low-dose propofol thalamus can explain cortical trough-max PAC (Fig 7A through 7D), a decrease in the overall level of background excitation would cause the thalamic UP states to exhibit sustained alpha while the DOWN states exhibit silent hyperpolarization, enabling peak-max PAC even at this lower dose regime. Thus, peak-max PAC is possible even under a very hyperpolarized thalamus with only moderate GABA_A_ potentiation. Tripling the GABA_A_ potentiation (high-dose) instead of doubling increased the probability of sustained alpha but could not account for a PAC shift under alternating SWO states by itself.

## Discussion

Human EEG studies have shown that, near the onset of and during LOC from propofol, the brain produces an alpha oscillation and a SWO [30]. These are coupled differently depending on the depth of anesthesia: at the onset or offset of LOC, the alpha rhythm appears in the trough of the SWO (“trough-max”), while at a deeper level of anesthesia, the alpha appears at the peak of the SWO (“peak-max”) [12]. In this study, we have used thalamic Hodgkin-Huxley-type simulations of the effects of propofol on g_H_, GABA_A_ conductance, and decay time, as well as changes in background excitation, to investigate how the thalamus can control such alpha-SWO PAC. We found that the hyperpolarized thalamus exhibited a novel thalamic sustained alpha rhythm that only occurs under GABA_A_ potentiation, as in propofol. Furthermore, depending on thalamic hyperpolarization level, the thalamus expressed alpha during either the corticothalamic SWO trough, producing trough-max output, or during the corticothalamic SWO peak, producing peak-max output.

The first central result is that the thalamus alone is able to produce a sustained alpha rhythm under potentiated GABA_A_ inhibition (Fig 2). Thus, we propose a novel thalamic rhythm not observed under normal/awake or sleep conditions, one that requires potentiation of GABA_A_ receptors. This sustained alpha is fundamentally different than native thalamic spindling due to its lack of waxing-and-waning; it lasted indefinitely, as long as our longest simulations (10 seconds). Secondly, this sustained alpha can be produced in the absence of oncoming SWO; this second finding extends the work of [28] by showing the how thalamus alone, independent of patterned cortical input, can robustly produce sustained alpha in the presence of propofol. The baseline model was able to produce spindling and lower-frequency rhythms, including delta and theta. However, at no level of depolarization, hyperpolarization, or g_H_ could it produce a sustained alpha rhythm (Fig 3). Only propofol potentiation of inhibition, by increasing GABA_A_ conductance and decay time, led to an ongoing alpha-frequency rhythm. The propofol-induced sustained alpha used the physiological building blocks of the spindling rhythm [25,37]. The mechanism was found to involve the T-current: the added RE cell inhibition led to greater de-inactivation of the T-current in the TC cells, leading to faster and more reliable TC bursts up to a sustained alpha frequency. With the potentiated inhibition, the sustained alpha appears in regions of parameter space where, in the baseline model without propofol, the network is spindling or silent and depolarized (Fig 4). Sustained alpha thus appears between regions of intrinsic oscillations and silent depolarization, created by TC cell membrane potential interactions with the T-current de-inactivation window. An interesting implication of these results is that direct application of propofol onto hyperpolarized thalamic circuits may be sufficient to produce sustained alpha. Since the thalamically-generated sustained alpha is expressed in the TC cells, which project to cortex, our models predict that the thalamic alpha could be the source of the coherent frontal alpha observed during anesthetic doses of propofol [11,30]. Our models further suggest that the thalamic alpha is generated independently of propofol-mediated changes to cortical or brainstem circuits, except for the necessary thalamic hyperpolarization. If this thalamic sustained alpha is consistently relayed to cortex, then we expect the oscillation to be detectable on the EEG. This prediction could be tested by infusion of propofol into the rodent thalamus alone, along with a hyperpolarizing agent, while recording cortical LFP or EEG and behavior. Importantly, such experiments may help further characterize the relative role of thalamic alpha in the production of unconsciousness.

Our results also suggest that alpha oscillations are the maximum intrinsic oscillatory frequency achievable by an independent, hyperpolarized thalamus, and this occurs only under GABA_A_ potentiation e.g. by propofol. The frequency of the ongoing oscillation increased with the conductance and/or decay time of inhibition but not beyond its sustained alpha peak at 300% of the baseline values, the values theoretically associated with high-dose propofol. This supports earlier computations in [28], which showed that, as the decay time increased to 300%, the frequency decayed to an asymptotic value. Increasing the GABA_A_ potentiation to 300% also led to a larger region of parameter space displaying sustained alpha, effectively increasing the probability of the rhythm occurring.

Our study suggests that the H-current effectively acts as a switch between the existence or absence of sustained alpha, and thus potentially as a switch between the conscious and unconscious states. This is because sustained alpha is possible only if the H-current falls below a threshold level. Note that the H-current is an intrinsic property of the TC cells and is separable from the level of hyperpolarization of the system. The thalamic H-current plays a permissive role in our simulations: intrinsic oscillations, including sustained ones, are possible only when g_H_ is adequately small, as in [37]. Such persistent activity is possible even when this conductance is almost zero, provided there is some depolarizing background excitation. The effect of propofol on thalamic H-currents is, however, controversial [38,40,41]. An H-current switch could be an important component of other anesthesias; sevoflurane has a very similar EEG profile to propofol [42], and may inhibit the thalamic H-current [43]. Conversely, this finding has important implications for potentially reversing the anesthetic state, suggesting that agents that increase the H-current in thalamic circuits will shift thalamic dynamics out of the region of sustained alpha oscillation and into the more depolarized awake/relay states. There are many neuromodulators of the H-current, including dopamine, norepinephrine, and serotonin [44], and these are widely known to be involved in the sleep-wake cycle; thus their H-current effects may affect consciousness.

Our models suggest that the level of thalamic hyperpolarization is the critical factor determining trough-max versus peak-max coupling as anesthetic dose is increased. Note that, while propofol GABA_A_ potentiation is necessary for sustained alpha to appear, changing the inhibition level is not sufficient for switching between PAC states. To achieve peak-max and trough-max coupling, we added another component to our model: cortical spiking, looking separately at thalamic dynamics under the influence of either UP or DOWN states coming from cortex [14,20]. Whereas DOWN state thalamus simply received no cortical input, the UP state thalamus received both a depolarization step, represented by an increase in background excitation, and cortical spiking. Of note, the UP and DOWN states were modeled the same for all levels of propofol, suggesting that trough-max and peak-max coupling could occur independently of changes to SWO properties. The trough-max coupling can be produced by introducing propofol GABA_A_ potentiation to a hyperpolarized thalamus during a cortical DOWN state, enabling a sustained alpha. The corresponding thalamic UP state is too depolarized to express alpha oscillations, so the alpha becomes phase-locked to the SWO trough in the cortical DOWN state. By contrast, peak-max coupling is possible from hyperpolarizing the thalamus more strongly (discussed below): the input from the corticothalamic UP state helps to compensate for the lower voltage level of the thalamus, enabling alpha oscillations only at the thalamic UP states. Thus, in peak-max, thalamocortical alpha oscillation occurs only during the peak of the cortical UP state, and there is no major thalamocortical signal during the cortical DOWN state. Since sustained alpha requires TC cell membrane potential interaction with the voltage-defined T-current window of de-inactivation, the thalamus can switch from trough-max to peak-max merely by becoming more hyperpolarized (and vice versa), even if the cortical activity remains the same.

Increasing thalamic hyperpolarization with propofol dose, an assumption of our model and a critical component of propofol-induced PAC, is supported by several lines of evidence. In our models, peak-max occurs during decreased, direct background excitation to thalamus that may result from the increased action of propofol on brainstem circuits at high doses [3,45,46]. Decreasing the background excitation term may represent potentiation of the potassium leak conductance (gKL) from those brainstem neuromodulatory effects, such as decreasing acetylcholine (ACh) output, increasing gKL, thus leading to hyperpolarization [47,48]. Recent modeling work has looked at the effects of varying gKL as a proxy for endogenous ACh and histamine (HA) changes in thalamic circuits, finding that spindle and SWO oscillations can be generated at certain levels of gKL [21]. Additionally, physostigmine, a cholinesterase inhibitor, has been found to reverse unconsciousness caused by propofol, ostensibly by enhancing ACh activity [49]. In the future, we plan to model propofol SWO directly, enabling better understanding of the PAC phenomenon and allowing us to understand the difference between general hyperpolarization and gKL on the thalamic and cortical systems.

This evidence points to the importance of propofol effects in both the thalamus and the brainstem for determining the PAC. Regardless of hyperpolarization level, there must be thalamic GABA_A_ potentiation from the propofol to enable the creation of sustained alpha oscillations in the thalamus. Similarly, even if there are sustained alpha oscillations, the thalamus will only exert different PAC regimes if brainstem neuromodulation dynamically hyperpolarizes the system. We initially thought that both of these changes would have similar effects, allowing us to model both GABA_A_ potentiation and hyperpolarization simultaneously, but this was not the case: the phasic component of GABA_A_ potentiation is critical to creating sustained alpha (see Fig 5).

The model suggests that trough-max alpha may be more coherent more than peak-max alpha, since trough-max alpha is intrinsically generated by the thalamus, whereas cortical input during peak-max may interfere with the thalamically generated alpha (Fig 8). During trough-max coupling, individual thalamic cells synchronize their sustained alpha bursts due to RE cell synchronization. This greater synchronization in trough-max coupling could increase alpha coherence in cortex and could partially explain the high frontal alpha coherence seen in trough-max [11,12,42]. The increased probability of sustained alpha firing in parameter space under peak-max alpha may account for the fact that peak-max alpha is found in more regions across the cortex than trough-max [11,12]. Further experimentation is needed to distinguish between the mechanisms behind such different alpha coherence in cortical circuits during trough-max and peak-max states.

This work has implications for other anesthetics and sedatives. Dexmedetomidine works in ways similar to sleep pathways by removing excitation to the thalamus and cortex [3,10,33]; experimentally, it produces spindling and SWO at higher doses [50] but not a strong, persistent alpha band that occurs in every SWO cycle. Our model explains the lack of sustained alpha with dexmedetomidine by the fact that it is not just thalamic hyperpolarization that is necessary but also the change in the time scale of the inhibition produced by GABA_A_ potentiation. Benzodiazepines, which do change the GABA_A_ time constant of inhibition, can produce beta or alpha oscillations depending on the dose [51,52]. Another GABA_A_ potentiator, sevoflurane also produces coherent frontal alpha and SWO simultaneously [42]. Recent sevoflurane experiments in rodents have shown that alpha oscillations in the thalamus lead the phase of those in the cortex [53], suggesting a thalamic source of alpha. Our model suggests that because sevoflurane induces GABA_A_ potentiation [54], probable thalamic hyperpolarization, and possible thalamic H-current inhibition [43], sevoflurane alpha could also be capable of trough-max and peak-max coupling to SWO.

Propofol-induced unconsciousness has been associated with SWO [4], alpha oscillations [9], and their PAC [11]. Our model predicts experimental manipulations that could dissociate alpha and PAC from the SWO component, delineating the role of each of these oscillations in the production of the unconscious state. Intracranial recordings have shown that humans experience LOC under propofol within seconds of SWO power manifesting [4], but trough-max PAC has also been found to occur immediately around LOC [11,12]. It is still unknown which, if any, of these spectral phenomena are causes rather than correlates of unconsciousness. It is possible that strong rhythmic synchronization of the thalamus alone, anywhere in the intrinsic oscillation range of SWO to sustained alpha, could be sufficient to disable consciousness. That is, unconsciousness could simply be the effect of disabling the relay function of the thalamus. In the case of propofol, sevoflurane, and similar anesthesias, the thalamocortical delivery of very coherent frontal alpha oscillations could actively disable higher-order thinking and possibly even arousal.

However, the relationship of PAC regimes to different levels of consciousness is still controversial [12,55]. Peak-max and trough-max do not just represent different doses of propofol [11], but also exhibit different levels of spatial coherence across oscillations [12]. Previous propofol PAC analysis [12]concluded: “…trough-max coupling of alpha amplitude with [low-frequency activity] phase was likewise concentrated at frontal electrodes…However, peak-max coupling dominated activity throughout frontal, temporal, and posterocentral regions” [12]. During trough-max, relay of propofol alpha to the frontal cortex specifically may interfere with self-awareness and other properties of consciousness, while still allowing for limited thalamocortical communication involving other parts of cortex. In this low level of unconsciousness immediately following LOC, a strong enough stimulus may be enough to retain or re-enable responsiveness. In fact, in a recent experiment [55], propofol-anesthetized patients were found to respond when experiencing a strongly noxious stimulus, but only when their EEG showed trough-max PAC or no PAC at all. In contrast, peak-max PAC may maintain a broadly disconnected state across the entire cortex, given both the higher power of its SWO oscillations and the broad distribution of peak-max PAC [12]. Since peak-max PAC disables responsiveness even under noxious stimuli [55], but SWO exists globally in both trough-max or peak-max, it may be that global alpha or PAC are the critical factors for disabling responsiveness and consciousness under peak-max PAC. Our model predicts that localized injection of propofol into a rodent along with hyperpolarizing the thalamus, possibly via localized injection of a gKL agonist, should cause strong thalamic and frontally coherent alpha to emerge, with or without SWO. If there is no accompanying SWO but the animal still loses consciousness, this would point to a clear, non-SWO, rhythmic way to modulate arousal that is used by propofol and sevoflurane.

Our models additionally suggest that propofol SWO may be generated from cortex. How propofol induces SWO is unknown, although both cortical and thalamic generation of SWO has been documented in normal sleep states [14,20,56]. In our model, intrinsic thalamic sustained alpha does not co-occur with lower frequency rhythms. This suggests that, if the anesthetized thalamus is dominated by sustained alpha, propofol SWO could be initiated exclusively by cortex. This is supported by the local, but not global, synchronization of propofol SWO in human intracranial data [4], as globally synchronous sleep SWO is thought to depend on thalamic relay of SWO [14,56]. This propofol SWO generation and its importance for modulating arousal could be tested by injecting propofol into only the cortex or the cortex and brainstem of a rodent. We predict that one would see propofol SWO, but no strong, sustained alpha; it is possible there would still be activity in the alpha range, however, representing endogenous spindles, owing to the brainstem-induced hyperpolarization of the thalamus.

Moving forward, modeling SWO directly is critical to understanding the PAC phenomena better. Many different mechanisms that have been found to successfully model slow waves in cortex [16,19–21,24,57] et al., and propofol slow waves may use an unknown, novel mechanism. The complexity of understanding propofol SWO generation was beyond the scope of this work, but in the future we plan to explore the many mechanisms possible for this, and how properties of these mechanisms could work in tandem with alpha mechanisms to produce propofol PAC characteristics.

Despite not modeling cortical SWO generation under propofol directly, we feel it is an interesting finding that our model thalamus can respond to cortical SWO with the appropriate phase amplitude coupling seen in increasing doses of propofol. This finding not only makes immediate experimental predictions, including that alpha may only be observed in subthreshold voltage traces of cortical neurons during trough-max, but it also provides constraints for how cortical UP and DOWN states of trough- and peak-max may be robust to thalamic alpha inputs.

We conclude that the thalamus may be the generator of this unique sustained alpha rhythm seen in propofol and the subcortical source of the cortically-detected alpha oscillation seen on EEG, and that propofol-induced PAC may be influenced by the specific effects of propofol on thalamic circuits. Further investigation based on this theory may enhance our understanding of the role of the thalamus and cortex for both anesthesia and consciousness in general.

## Materials and methods

### Model design: Thalamus

**Fig 9:**
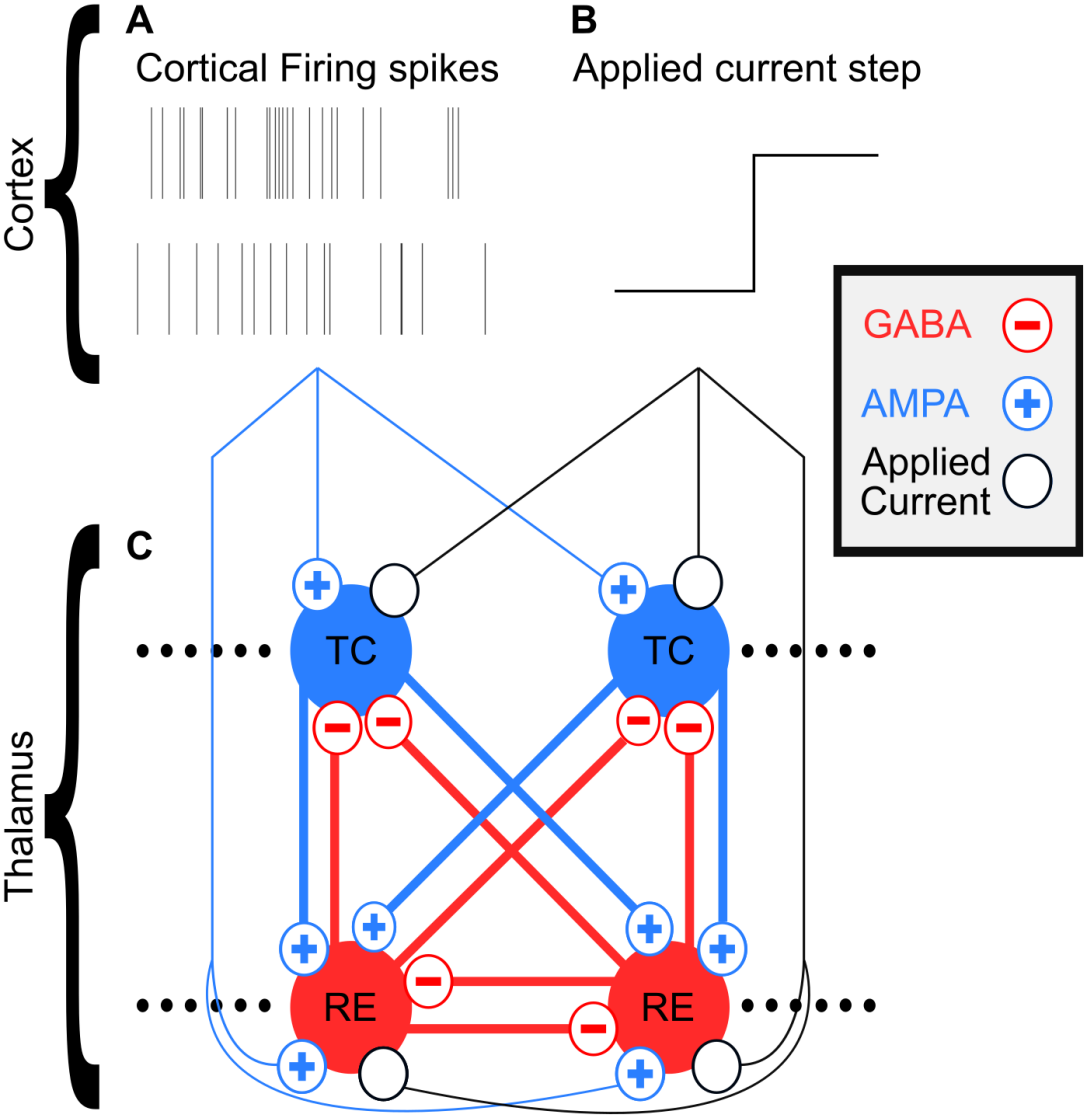
Diagram illustrating thalamic network model and its cortical inputs. (A) Example 12 Hz Poisson process-generated spiketrains used for corticothalamic UP state spikes. (B) Example background excitation/applied current step used for corticothalamic UP state. (C) Model network of thalamus, its connections, and its inputs from cortex.

Our model is almost identical to the thalamic model used in [28], which is based on [37]. Our model network is illustrated in Fig 9C. The only nontrivial changes were differences in the size of the network and our inclusion of a weak RE to TC GABA_B_ current, but these do not dramatically change the behavior of the system. All of our simulations used 50 TC and 50 RE Hodgkin-Huxley single-compartment cells connected all-to-all. See the S1 Appendix for all the equations and their baseline parameterizations.

As in [28], we used a relatively high gKL to simulate a baseline hyperpolarized thalamus. While many neuromodulators act on gKL [47,48], we explored the impact of directly varying background excitation instead. Modeling the effect of background excitation instead of gKL lets us more broadly model uncharacterized brainstem input such as neuromodulators, second-order effects from neuromodulators, and loss of brainstem excitation. Using background excitation also lets us model these hyperpolarizing changes in a voltage-invariant way, unlike gKL with its equilibrium potential. Our resulting thalamic states across all g_H_-background excitation planes and simulations therefore represent a thalamus hyperpolarized from any awake/relay state. If our thalamus was not this hyperpolarized, it would have responded more strongly to cortical spiking input in our simulations under CF in Fig 7A and 7E.

Baseline (no propofol) simulations used all default parameters as listed in the S1 Appendix. Low-dose propofol was modeled by doubling g_**GABAA**_ and τ_**GABAA**_ for all GABA_A_ synapses in the model, while high-dose propofol was modeled by tripling g_**GABAA**_ and τ_**GABAA**_, as in [8,28]. This parametrization was derived in two steps, as mentioned in the subsection Methods > Computational Model > Propofol in [8]. First, we independently calculated the EC50 propofol concentration in human blood to be ~0.38 uM using the known propofol blood plasma concentration [1], the molecular weight of propofol (178.271 g/mol), and the percentage of free propofol in plasma [58]. This value was roughly the same as what others calculated, 0.4 uM [59]. We also calculated the high-dose concentration to be ~1.0 uM using [60]. Having derived the concentrations, we then used experimental data from propofol IPSC effects [60] to predict the percent increase of g_**GABAA**_ and τ_**GABAA**_ at these doses. According to these calculations [61], low-dose propofol at a concentration of 0.4 uM should cause an increase in g_**GABAA**_ and τ_**GABAA**_ to 200% of baseline, and high-dose propofol at 1.0 uM should cause an increase to 300% of baseline. We are aware that there are other *in vitro* experiments that come to different conclusions about the magnitude of these propofol effects [62]. However, we believe the experiments used in our calculations [61] best represent the state of our hyperpolarized, anesthetized thalamic circuitry [61].

Only by investigating the thalamus in isolation from cortex could we show it produces oscillations independent of cortical input. In our prior work modeling the entire thalamocortical loop [28,29], the mechanistic source of the propofol-induced alpha oscillation is not examined in detail, and part of the purpose of the present study was to investigate whether thalamus could be a generator of the thalamocortical alpha observed in these previous models. We are confident that this thalamic alpha generation will not be completely eliminated by the introduction of responsive cortical cells, since cortical inputs were shown to reliably output alpha oscillations under anesthetic conditions previously [28]. In fact, the main point of [28] was that thalamocortical interaction enhanced propofol alpha under feedback, instead of eliminating it.

Finally, while 50 cells of each thalamic cell type may seem too small to be effective, even a thalamic population of this size can directly affect the activity of many cortical cells. By one measure [63], 50 thalamic cells may be enough to connect to roughly 8000 cortical cells at a 1–160 thalamocortical-cortical cell ratio. Furthermore, the ratio between higher-order thalamic cells and their downstream cortical cell targets could be substantially higher, but is unclear [64]. Furthermore, since we do not model cortical cells directly, we do not claim to model the EEG signal directly. In our prior work modeling the entire thalamocortical loop [28,29], we do simulate a cortically-generated EEG signal, finding that our prior model produces the expected propofol phenomena even when the thalamic network is substantially smaller than in the current work. We believe these reasons are sufficient evidence that we do not need to simulate very many thalamic cells in order to accurately model both the general thalamic output to the cortex and the resulting EEG signal from the cortex.

### Model design: Cortex

We did not model cortex directly; instead, we chose to model its inputs to the thalamus across the SWO cycle artificially. Corticothalamic UP states were modeled as a combination of AMPAergic cortical firing generated from a 12 Hz Poisson process [28], shown in Fig 9A, and stepwise increases in background excitation, shown in Fig 9B. Corticothalamic DOWN states were modeled as the absence of these inputs. These individual components of corticothalamic impact during SWO phases are well supported [14,20] and allowed us to study their influences on the system independently of each other.

We chose to model only UP and DOWN thalamic steady states instead of transitions due to the high number of degrees of freedom in the waveform of a dynamic transition. In our investigations, we decided it was too complex to compare all the different possible UP and DOWN states in the thalamus robustly at the same time we were varying transition attributes. We decided to characterize the steady states of the system before moving on to dynamic state changes in the future. Furthermore, as we move forward to model the slow waves in cortical cells directly, this will allow us to examine their thalamocortical interaction more accurately than by modeling artificial signals, since artificial signals would not allow for responsive cortical cell changes to the oscillation. Additionally, cortically-generated SWO will offer greater modeling constraints than the freedom of artificial signals, requiring less simulation.

### Behavior regime classification

In Figs 3, 4, 5 and 7, we hand-classified the behavior regime of each simulation (represented by an individual pixel) across the g_H_-background excitation plane. By examining the voltage traces of the TC cells, we classified simulations into the following list of behavior regimes: nonphysiological, silent/hyperpolarized, transients/spindle oscillations, sub-alpha oscillations, alpha oscillations, or silent/depolarized. Nonphysiological (black) represented simulations where the average membrane potential was either hyperpolarized to be less than -100 mV, depolarized to be greater than -50 mV, or depolarized and firing pathologically rapidly; “pathologically rapidly” was defined as persistent firing of the same frequency purely in response to extreme depolarization of the system, and it only occurred under extreme depolarization at the limits of the system. Silent/hyperpolarized (grey), shown in Fig 3C, represented states where the TC cells did not intrinsically fire but their average membrane potential was above -100 mV and below the “T -current window” from roughly -72 mV to -80 mV. Transients/spindle oscillations, shown in Fig 3D, were defined as thalamic states where the TC cells either expressed waxing-and-waning spindles oscillations (the spindles themselves were around 10 Hz), or transients that lasted long enough that the system could possibly wax into a spindle past the 8 seconds of simulation performed. Sub-alpha oscillations, shown in Fig 3E, were defined as states where the TC cells expressed persistent oscillations that lasted either most or all of the 8 second long simulations, and the oscillations were less than the alpha range of 8 to 13 Hz; different simulations in this category expressed firing in slow (0.1–1.5 Hz), delta (1.5–4 Hz), or theta (4–8 Hz) frequency ranges. Alpha oscillations were defined as those that had TC cells bursting at 8–13 Hz for the entire duration of the 8 second long simulations; they were differentiated from transient/spindling oscillations by the lack of wax-and-waning as described in the Results. Silent/depolarized states, shown in Fig 3F, were defined as those that had TC cells not firing and had an average membrane potential depolarized above the T-current window but below -50 mV.

### Simulations and reproducibility

Simulations were run using the open source DynaSim MATLAB toolbox [65] originally created by Jason Sherfey, on the Massachusetts Green High Performance Computing Center. Mechanism files used to populate the model in DynaSim are freely available on GitHub [66], and code to reproduce all simulations and preliminary figures is also available on GitHub [67] (except for MATLAB itself).

## Supporting information

S1 Appendix Equations for computational models. (PDF)
