# Supplementary material for: Thalamocortical control of propofol phase-amplitude coupling
Source: PLoS Comput Biol. 2017 Dec 11;13(12):e1005879. doi: 10.1371/journal.pcbi.1005879 (PMC5739502; doi:10.1371/journal.pcbi.1005879)
Supplement: S1 Appendix — (PDF) [file pcbi.1005879.s001.pdf]

# Supplemental Appendix 1 for "Thalamocortical control of propofol phase-amplitude coupling": Equations

Austin Soplata

May 19, 2017

## 1 General Notes

Units of maximal conductances and currents for all those below are in $\frac{mS}{cm^2}$ and $\frac{\mu A}{cm^2}$, respectively, and are from [Hodgkin and Huxley, 1952], [Destexhe et al., 1993], and [Destexhe et al., 1996] by way of [Ching et al., 2010] unless otherwise indicated. All simulations were done with 50 TC and 50 RE cells, and for at least 6 seconds, solved using Euler integration, with a time resolution (dt) of 0.01 ms.

## 2 Intrinsic TC Cell Equations

### 2.1 Voltage / Membrane Potential

$$\dot{V}_{TC} = I_{applied} - I_{Na} - I_K - I_T - I_H - I_{Leak} - I_{KLeak} - I_{syn(\rightarrow TC)}$$

$I_{applied}$ was used to model background excitation (aka "applied current"), and was therefore variable, depending on the simulation. The bounds we investigated were roughly -2 to 2 $\frac{uA}{cm^2}$. $I_{applied}$ was always applied identically to TC and RE cells.

### 2.2 Sodium Channel Current

$$I_{Na} = \bar{g}_{Na} m^3 h (V_T - E_{Na})$$

Parameters:

$V_T = V + 35mV$

$\bar{g}_{Na} = 90 \frac{mS}{cm^2}$

$E_{Na} = 50mV$

State Variable Equations: m, h

$\dot{m} = \alpha_m \cdot (1-m) - \beta_m \cdot m$

$\alpha_m = \frac{0.32 \cdot (13 - V_T)}{exp(\frac{13-V_T}{4})-1}$

$\beta_m = \frac{0.28 \cdot (V_T - 40)}{exp(\frac{V_T-40}{5})-1}$

$\dot{h} = \alpha_h \cdot (1-h) - \beta_h \cdot h$

$\alpha_h = 0.128 \cdot exp(\frac{17-V_T}{18})$

$\beta_h = \frac{4}{1+exp(\frac{40-V_T}{5})}$

### 2.3 Potassium Channel Current

$$I_K = \bar{g}_K n^4 (V_T - E_K)$$

Parameters:

$V_T = V + 25mV$

$\bar{g}_K = 10 \frac{mS}{cm^2}$

$E_K = -100mV$

State Variable Equations: n

$\dot{n} = \alpha_n \cdot (1-n) - \beta_n \cdot n$

$\alpha_n = \frac{0.032(15-V_T)}{exp(\frac{15-V_T}{5})-1}$

$\beta_n = 0.5 exp(\frac{10-V_T}{40})$

### 2.4 T-type Calcium Current (T-current)

$$I_T = \bar{g}_T m_\infty^2 h (V_T - E_T)$$

Parameters:

$V_T = V + 2mV$

$\bar{g}_T = 2 \frac{mS}{cm^2}$

$E_T = 1000 \frac{8.31441 * 309.15}{2 * 96486} ln \frac{2}{[Ca]_i}$

State Variable Equations: m

$m_\infty = \frac{1}{1+exp(\frac{-(V_T+57)}{6.2})}$

State Variable Equations: h

$\dot{h} = \frac{h_\infty - h}{\tau_h}$

$h_\infty = \frac{1}{1+exp(\frac{V_T+81}{4})}$

$\tau_h = \left(30.8 + \frac{211.4 + exp(\frac{V_T+113.2}{5})}{1+exp(\frac{V_T+84}{3.2})}\right)/3.73$

State Variable Equations: $[Ca]_i$

$\dot{[Ca]}_i = max\left(\frac{-10 \cdot I_T}{2 \cdot 96489}, 0\right) + \frac{0.00024 - [Ca]_i}{5}$

### 2.5 Hyperpolarization-activated Current (H-current)

$$I_H = \bar{g}_H (o_1 + 2(1 - c_1 - o_1))(V - E_H)$$

Note: This is the more complex [Destexhe et al., 1996] formulation of the H-current, NOT that of [Destexhe et al., 1993].

Parameters:

$\bar{g}_H = 0.025 \frac{mS}{cm^2}$ (but it depends... ask me)

$E_H = -43mV$

Like $I_{applied}$, $\bar{g}_H$ changes depending on the simulation under investigation. See Results for more detail.

State Variable Equations: $o_1, p_0, c_1$

$\dot{o}_1 = k_4(1 - c_1 - o_1) - (k_{3,p_0} \cdot o_1)$

$\dot{p}_0 = k_2(1 - p_0) - (k_{1,Ca} \cdot p_0)$

$\dot{c}_1 = \beta \cdot o_1 - \alpha \cdot c_1$

$k_{1,Ca} = k_2 \cdot \left(\frac{[Ca]_i}{[Ca]_{crit}}\right)^4$

- $[Ca]_i$ is a state variable determined by the T-current.

$k_2 = 0.0004$

$k_{3,p_0} = k_4 \cdot \left(\frac{1-p_0}{0.01}\right)^1$

$k_4 = 0.001$

$[Ca]_{crit} = 0.002$ (mM of Calcium)

$\alpha = \frac{h_\infty}{\tau_s}$

$\beta = \frac{1-h_\infty}{\tau_s}$

$h_\infty = \frac{1}{1+exp(\frac{V+75}{5.5})}$

$\tau_s = \left(20 + 1000/\big(exp(\frac{V+71.5}{14.2}) + exp(\frac{-(V+89)}{11.6})\big)\right)/1$

## 2.6 Leak Currents

$$I_{Leak} = \bar{g}_{Leak}(V - E_{Leak})$$

Parameters:

$\bar{g}_{Leak} = 0.01\frac{mS}{cm^2}$

$E_{Leak} = -70mV$

$$I_{KLeak} = \bar{g}_{KLeak}(V - E_{KLeak})$$

Parameters:

$\bar{g}_{KLeak} = 0.0172\frac{mS}{cm^2}$

$E_{KLeak} = -100mV$

# 3 Intrinsic RE Cell Equations

## 3.1 Voltage / Membrane Potential

$$\dot{V}_{RE} = I_{applied} - I_{Na} - I_K - I_T - I_{Leak} - I_{KLeak} - I_{syn(\to RE)}$$

$I_{applied}$ was used to model background excitation (aka "applied current"), and therefore was variable, depending on the simulation. The bounds we investigated were roughly -2 to 2 $\frac{uA}{cm^2}$. $I_{applied}$ was always applied identically to TC and RE cells.

## 3.2 Sodium Current

$$I_{Na} = \bar{g}_{Na}m^3h(V_T - E_{Na})$$

Parameters:

$V_T = V + 55mV$

$\bar{g}_{Na} = 200\frac{mS}{cm^2}$

$E_{Na} = 50mV$

State Variable Equations: m, h

$\dot{m} = \alpha_m \cdot (1 - m) - \beta_m \cdot m$

$\alpha_m = \frac{0.32\cdot(13-V_T)}{exp(\frac{13-V_T}{4})-1}$

$\beta_m = \frac{0.28\cdot(V_T-40)}{exp(\frac{V_T-40}{5})-1}$

$\dot{h} = \alpha_h \cdot (1 - h) - \beta_h \cdot h$

$\alpha_h = 0.128 \cdot exp(\frac{17-V_T}{18})$

$\beta_h = \frac{4}{1+exp(\frac{40-V_T}{5})}$

## 3.3 Potassium Current

$$I_K = \bar{g}_K n^4(V_T - E_K)$$

Parameters:

$V_T = V + 55mV$

$\bar{g}_K = 20\frac{mS}{cm^2}$

$E_K = -100mV$

State Variable Equations: n

$\dot{n} = \alpha_n \cdot (1 - n) - \beta_n \cdot n$

$\alpha_n = \frac{0.032(15-V_T)}{exp(\frac{15-V_T}{5})-1}$

$\beta_n = 0.5exp(\frac{10-V_T}{40})$

## 3.4 T-type Calcium Current (T-current)

$$I_T = \bar{g}_T m^2h(V_T - E_T)$$

Parameters:

$V_T = V + 4mV$

$\bar{g}_T = 3\frac{mS}{cm^2}$

$E_T = 120mV$

State Variable Equations: m

$\dot{m} = \frac{m_\infty - m}{\tau_m}$

$m_\infty = \frac{1}{1+exp(\frac{-(V_T+50)}{7.4})}$

$\tau_m = \left(3 + \frac{1}{exp(\frac{V_T+25}{10})+exp(\frac{-(V_T+100)}{15})}\right)/\phi_m$

$\phi_m = 6.81$

State Variable Equations: h

$\dot{h} = \frac{h_\infty - h}{\tau_h}$

$h_\infty = \frac{1}{1+exp(\frac{V_T+78}{5})}$

$\tau_h = \left(85 + \frac{1}{exp(\frac{V_T+46}{4})+exp(\frac{-(V_T+405)}{50})}\right)/\phi_h$

$\phi_h = 3.73$

State Variable Equations: $[Ca]_i$

$[\dot{Ca}]_i = max\left(\frac{-10 \cdot I_T}{2 \cdot 96489}, 0\right) + \frac{0.00024-[Ca]_i}{5}$

### 3.5 Leak Currents

$$I_{Leak} = \bar{g}_{Leak}(V - E_{Leak})$$

Parameters:

$\bar{g}_{Leak} = 0.05\frac{mS}{cm^2}$

$E_{Leak} = -90mV$

## 4 Synaptic Equations

### 4.1 Summary Equations and Connectivity

$$-I_{syn(\to TC)} = -I_{GABA_A(RE\to TC)} - I_{GABA_B(RE\to TC)} - I_{Poisson(CT\to TC)}$$

$$-I_{syn(\to RE)} = -I_{AMPA(TC\to RE)} - I_{GABA_A(RE\to RE)} - I_{Poisson(CT\to RE)}$$

$TC \to RE$, $RE \to TC$, and $RE \to RE$ connections were all-to-all. $CT \to TC$ and $CT \to RE$ connections were all-to-all, but with a 50% connection probability. Note that there are not true cortical (CT) cells we are explicitly modeling, but rather we are only modeling artificial spiketrains going to the truly Hodgkin-Huxley modeled TC and RE cells.

### 4.2 AMPA Current

$$I_{AMPA} = \frac{\bar{g}_{AMPA}}{N_{pre}} s_{AMPA}(V_{post} - E_{AMPA})$$

Parameters:

$\bar{g}_{AMPA(TC\to RE)} = 0.08\frac{mS}{cm^2}$

$E_{AMPA} = 1mV$

$N_{pre}$ = number of presynaptic cells

Note that the canonical $E_{AMPA}$ is usually 0 mV, however we used 1 mV due to a bug in the code at the time. This difference does not change our results.

State Variable Equations: $s_{AMPA}$

$\dot{s}_{AMPA} = 5(1 + tanh(\frac{V_{pre}}{4}))(1 - s_{AMPA}) - \frac{s_{AMPA}}{\tau_{AMPA}}$

$\tau_{AMPA} = 2ms$

### 4.3 $GABA_A$ Current

$$I_{GABA_A} = \frac{\bar{g}_{GABA_A}}{N_{pre}} s_{GABA_A}(V_{post} - E_{GABA_A})$$

Parameters:

$\bar{g}_{GABA_A(RE\to TC)} = 0.069\frac{mS}{cm^2}$

$\bar{g}_{GABA_A(RE\to RE)} = 0.069\frac{mS}{cm^2}$

$E_{GABA_A} = -80mV$

$N_{pre}$ = number of presynaptic cells

State Variable Equations: $s_{GABA_A}$

$\dot{s}_{GABA_A} = 2(1 + tanh(\frac{V_{pre}}{4}))(1 - s_{GABA_A}) - \frac{s_{GABA_A}}{\tau_{GABA_A}}$

$\tau_{GABA_A} = 5ms$

Note that for "low-dose" propofol, we multiply $\bar{g}_{GABA_A}$ and $\tau_{GABA_A}$ by 2, and for "high-dose" propofol, we multiply $\bar{g}_{GABA_A}$ and $\tau_{GABA_A}$ by 3.

### 4.4 $GABA_B$ Current

$$I_{GABA_B} = \frac{\bar{g}_{GABA_B}}{N_{pre}} \frac{g^4}{g^4 + 100}(V_{post} - E_{GABA_B})$$

Note: the maximal synaptic conductance, $\bar{g}_{GABA_B}$, is different from the state variable of $GABA_B$ named $g$. Apologies for the poor naming, but this naming is inherited and used for historical consistency.

Parameters:

$\bar{g}_{GABA_B(RE\to TC)} = 0.001\frac{mS}{cm^2}$

$E_{GABA_B} = -95mV$

$N_{pre}$ = number of presynaptic cells

State Variable Equations: r,g

$\dot{r} = k_1(2(1 + tanh(\frac{V_{pre}}{4}))(1 - r) - k_2 \cdot r$

$\dot{g} = k_3 \cdot r - k_4 \cdot g$

$k_1 = 0.5(mM^{-1}ms^{-1})$

$k_2 = 0.0012(ms^{-1})$

$k_3 = 0.18(ms^{-1})$

$k_4 = 0.034(ms^{-1})$

This is, very slightly, an original formulation of the $GABA_B$ current. The state variable $r$ for $GABA_B$ is 'customized' here, in that, rather than the popular formulation of a 0.5 mM box 0.3 ms long for the transmitter amount, inherited from [Destexhe et al., 1996], the VERY similar $2(1+tanh(\frac{V_{pre}}{4}))$ method of calculating transmitter concentration from [Olufsen et al., 2003] is used. 0.3 ms is about as long as a neuron's voltage

is above 0 mV, the latter being the definition of when this voltage-sensitive transmitter concentration is non-zero. The same concentration amplitude was used since I had already seen a long time of realistic results with $GABA_A$ responding to it, and $GABA_B$'s effect obviously has a much more malleable effector in its maximal conductance. In many ways, this is a simplified version of the $GABA_B$ current from [Vijayan and Kopell, 2012]: instead of a fixed spike of transmitter concentration when the presynaptic cell spikes, we use the same GABA concentration calculation method from the $GABA_A$ current.

### 4.5 Artificial Poisson Cortical Spikes

These cortical spikes were made to simulate AMPAergic spikes to thalamic populations. They were adapted from the double-exponential synaptic model in Chapter 6 of [De Schutter, 2009]. Note that we are not explicitly modeling cortical cells, but instead creating our own spiketrains.

$$I_{Poisson} = \frac{\bar{g}_{Poisson}}{N_{pre}} G_e(t)(V_{post} - E_{AMPA})$$

| Parameters: |
| --- |
| $\bar{g}_{Poisson(CT \to TC)} = 0.05 \frac{mS}{cm^2}$ |
| $\bar{g}_{Poisson(CT \to RE)} = 0.05 \frac{mS}{cm^2}$ |
| $E_{GABA_B} = 1mV$ |
| $N_{pre}$ = number of presynaptic cells (accounting |
| for 50% connection probability) |

Note: due to an error in normalization of the spike generation function, the **effective** synaptic strength of these spikes is tripled, meaning the final $\bar{g}_{Poisson}$ that the thalamic cells ACTUALLY see are closer to 0.15 $\frac{mS}{cm^2}$. All equations and parameters are shown AS RUN to maintain accuracy, but the normalization should be redone if one wants to use this Poisson AMPA functionality.

| Functions: |
| --- |
| $G_e(t) = \text{spiketrain} \cdot \frac{10}{2-0.5}(exp(\frac{-max(t-1,0)}{2}) - exp(\frac{-max(t-1,0)}{0.5}))$ |

$G_e$ is the convolved spiketrain precalculated prior to running the simulation; the relevant data to the current step pulled from the vector and used in the current calculation at each step. The spiketrain is calculated by taking a matrix of uniformly distributed data, removing all values except for those as rare as how frequently a 12 Hz spike would happen in proportion to the entire time length. The final $G_e$ will be a convolved spiketrain that is random between each "source cell" spiketrain.

## 5 Reproducibility and Code

All final simulations were run using the DynaSim software package [Sherfey, 2016]. The individual mechanism files for use with DynaSim are available online [Soplata, 2017b], as is a companion download of DynaSim itself along with the mechanisms where the only thing you need is a copy of MATLAB, [Soplata, 2017a]

# References


S. Ching, A. Cimenser, P. L. Purdon, E. N. Brown, and N. J. Kopell. Thalamocortical model for a propofol-induced alpha-rhythm associated with loss of consciousness. *Proceedings of the National Academy of Sciences*, 107(52):22665–22670, December 2010. ISSN 0027-8424, 1091-6490. doi: 10.1073/pnas.1017069108. URL `http://www.pnas.org/cgi/doi/10.1073/pnas.1017069108`.

Erik De Schutter, editor. *Computational Modeling Methods for Neuroscientists*. The MIT Press, September 2009. ISBN 978-0-262-01327-7. URL `http://mitpress.universitypressscholarship.com/view/10.7` DOI: 10.7551/mitpress/9780262013277.001.0001.

A Destexhe, A Babloyantz, and T J Sejnowski. Ionic mechanisms for intrinsic slow oscillations in thalamic relay neurons. *Biophysical Journal*, 65(4): 1538–1552, October 1993. ISSN 0006-3495. URL `http://www.ncbi.nlm.nih.gov/pmc/articles/PMC1225880/`.

Alain Destexhe, Thierry Bal, David A. McCormick, and Terrence J. Sejnowski. Ionic mechanisms underlying synchronized oscillations and propagating waves in a model of ferret thalamic slices. *Journal of Neurophysiology*, 76(3):2049–2070, 1996.

Alan L. Hodgkin and Andrew F. Huxley. A quantitative description of membrane current and its application to conduction and excitation in nerve. *The Journal of physiology*, 117(4):500, 1952. URL `http://www.ncbi.nlm.nih.gov/pmc/articles/pmc1392413/`.

Mette S. Olufsen, Miles A. Whittington, Marcelo Camperi, and Nancy Kopell. New roles for the gamma rhythm: population tuning and preprocessing for the beta rhythm. *Journal of Computational Neuroscience*, 14(1):33–54, 2003. URL `http://link.springer.com/article/10.1023/A:1021124317706`

Jason Sherfey. DynaSim: a Matlab toolbox for rapidly building and exploring neural models. 2016.

Austin Soplata. propofol-coupling-2017-full, 2017a. URL `https://github.com/asoplata/propofol-coupling-2017-full`.

Austin Soplata. propofol-coupling-2017-mechanisms, 2017b. URL `https://github.com/asoplata/propofol-coupling-2017-mecha`

S. Vijayan and N. J. Kopell. Thalamic model of awake alpha oscillations and implications for stimulus processing. *Proceedings of the National Academy of Sciences*, 109(45):18553–18558, November 2012. ISSN 0027-8424, 1091-6490. doi: 10.1073/pnas.1215385109. URL `http://www.pnas.org/cgi/doi/10.1073/pnas.1215385109`.
